# The Good Wishes Project: An End-of-Life Intervention for Individuals Experiencing Homelessness

**DOI:** 10.1089/pmr.2020.0006

**Published:** 2020-11-18

**Authors:** Alissa Tedesco, Leslie Shanks, Naheed Dosani

**Affiliations:** Inner City Health Associates, Toronto, Ontario, Canada.

**Keywords:** marginalized populations, palliative care, underserved populations (homeless poverty stricken)

## Abstract

***Background:*** Individuals experiencing homelessness face marginalization, dehumanization, and barriers to accessing quality palliative care. Inspired by the 3 Wishes Project, the Good Wishes Project (GWP) facilitates granting wishes to individuals experiencing homelessness and receiving palliative care with a goal of enhancing comfort and personalizing the end-of-life experience.

***Objective:*** The main objective of this study was to elicit provider perspectives on the utility of the GWP in the delivery of end-of-life care to a population of homeless and vulnerably housed individuals.

***Design:*** For this qualitative study, GWP client information and wish data were collected anonymously and analyzed quantitatively and descriptively. Semistructured interviews were conducted with health and social service professionals who cared for GWP clients. Interviews were recorded, transcribed, and analyzed through qualitative content analysis.

***Results:*** At the time of evaluation, there were a total of 27 clients in the GWP. At 14 months after the project's launch, 40 wishes had been made, 24 of which had been granted. Wishes were classified into five categories: basic necessities, end-of-life preparations, personal connections, paying-it-forward, and leisure. From the provider perspective (*n* = 7), the project was found to have utility in three main domains: establishing and enhancing connection, satisfying basic needs, and promoting person-centered care.

***Conclusions:*** The GWP is a promising psychosocial intervention in providing quality palliative care to individuals experiencing homelessness, whose lives have largely been burdened with hardship and marginalization.

## Background

Persons experiencing homelessness suffer from high rates of morbidity and mortality as compared with the general population.^1–13^ Despite their complex needs, this population faces poor access to quality palliative care services.^9,14–21^ Currently, there is sparse literature on the end-of-life needs of persons experiencing homelessness. What is available emphasizes that individuals experiencing homelessness have end-of-life concerns that are often distinct from the general population.^22^ For a group that has largely been marginalized with often limited social support, a common fear is dying anonymously,^23^ which illustrates the “profound loss of self” associated with homelessness.^24^ Compounded by the belief that their care will be poor at end of life,^25^ this is a clear call for health care professionals to practice “dignity-conserving care”^26^ for individuals experiencing homelessness, particularly at end of life. This type of care is rooted in “affirming the patient's value—that is, seeing the person they are now or were, rather than just the illness they have”.^26^

The 3 Wishes Project (3WP),^27^ an intervention embedded in a mixed-methods study, provides an example of dignity-conserving care. The authors aimed to humanize the intensive care unit (ICU) setting by eliciting wishes from dying patients, their families, and clinicians, aimed at honoring each patient. In doing so, they found the project was able to personalize the dying process in the ICU in three ways: dignifying patients and celebrating their lives, giving the family a voice and creating positive memories, and fostering clinician compassion and humanity. The project has since been successfully replicated in >20 ICUs in North America.^28^ Inspired by the 3WP, the Good Wishes Project (GWP) was created as a partnership between the Inner City Health Associates' (ICHA) Palliative Education and Care for the Homeless (PEACH) program and Haven Toronto. It is funded by the Sovereign Order of St. John of Jerusalem, Knights Hospitaller. The program grants wishes to individuals experiencing homelessness with the goal of enhancing comfort and personalizing the end-of-life experience.

## Objectives

The main objective of this study was to elicit provider perspectives on the utility of the GWP in the delivery of end-of-life care to individuals experiencing homelessness or vulnerable housing. The secondary objective of this study was to determine the barriers and facilitators of adapting the 3WP to this population and develop recommendations to address them.

## Methods

This qualitative study was conducted in Toronto. The GWP began enrolling clients in June 2016. This study began 14 months after the GWP inception, in August 2017, and was completed in May 2018. The study protocol was approved by the St. Michael's Hospital Research Ethics board (Study ID 17-149).

### GWP description

The inclusion criteria for the GWP were (1) client of the PEACH program and (2) have an estimated prognosis of <12 months. A PEACH team member identified eligible clients and introduced the project to the client. Informed consent for referral to the GWP was obtained. The GWP team comprising staff from Haven Toronto then met with the clients to elicit up to three wishes, often with the assistance of the care team. The decision making on granting wishes was done by the GWP team. There were no fixed criteria for acceptable wishes, but a rough guideline of a financial limit of $200 to 300 per individual was used. The GWP team was responsible for executing the wishes. In many cases however, this was coordinated with the assistance of the care team. The project was executed without any additional staff resources but did require additional funding for the wishes.

### Data collection on clients and wishes

Anonymized information on GWP clients was provided in aggregate to the research team by the GWP/PEACH team. Data included the number of GWP clients, their age, gender, and palliative diagnosis. In addition, wishes, both granted and not granted, were provided.

### Recruitment and interviews

Care team members comprising palliative clinicians from PEACH and other health and social service providers were identified through purposive sampling to select those who had significant interaction with GWP clients. In addition, members of the GWP team were sampled. Potential participants were invited for an interview by A.T. after consenting to be contacted. Several perspectives were obtained by sampling varied provider groups in terms of profession, work setting, and experience. Interviews were conducted to explore participants' experience with the GWP, and its successes and challenges as a program. The semistructured interview guide is provided in Supplementary Appendix SA1. The interviews were conducted by A.T. and were 20 to 45 minutes in duration. Participants were sent a letter of appreciation at the end of the study.

### Analysis

Interviews were audio recorded, transcribed verbatim, and deidentified by a member of the research team (A.T.). A fidelity check was performed by reviewing the transcripts while listening to audio recordings. The transcripts were analyzed by one investigator (A.T.) through inductive content analysis, where codes were derived directly from the data.^29^ An initial list of codes was developed through open coding. As data collection proceeded, these codes were updated and refined. Similar codes were then grouped into categories. These categories were used to develop central themes. Data saturation was deemed to be reached when no new themes or codes emerged and no further interviews were needed.^30^ Wishes were interpreted and classified into categories based on theme or type of gift.

## Results

### Clients and wishes

At the time of data collection, the GWP had enrolled 27 clients. The mean age was 59.3 (SD 11.1) years and was predominantly male (74.1%). Most clients had malignancy as their primary palliative diagnosis. Client demographics are outlined in Table 1.

**Table 1.** Good Wishes Project Client Demographics (*n* = 27)

Age (years)	Mean 59.3 (SD 11.1)
Gender, *n* (%)	
Male	20 (74.1)
Female	7 (25.9)
Other	0
Primary palliative diagnosis, *n* (%)	
Malignancy	17 (62.9)
Cirrhosis	2 (7.4)
Chronic obstructive pulmonary disease	5 (18.5)
Peripheral vascular disease	1 (3.7)
Chronic kidney disease	1 (3.7)
Unknown	1 (3.7)

At the end of the evaluation period, a total of 40 wishes were made, 24 of which had been granted. Nine wishes remained pending and seven were not completed (either deemed inappropriate or not able to be completed due to death or difficulty of implementation). Number of granted wishes ranged from 0 to 3 per client. Implemented wishes were classified into five categories: basic necessities, end-of-life preparations, personal connections, paying-it-forward, and leisure (details given in Table 2).

**Table 2.** Examples of Wishes Implemented, by Category

Basic necessities
Clothing
Home appliances (e.g., air conditioner)
Personal health devices (e.g., glasses)
Groceries
Rent
End-of-life preparations
Funeral planning
Ambulance fees
Personal connections
Phone bills
Visits with family
Meals with family/friends/providers
Paying-it-forward
Gift for friend
Donation to a charitable organization
Leisure
Electronic device (e.g., tablet)
Musical instrument (e.g., guitar)

### Provider interviews

Eight individuals involved in the care of GWP clients were approached to participate in this study. One person declined due to unknown reasons. Seven participants consented to be interviewed, which included two nurses (28.6%), two care coordinators (28.6%), two social workers (28.6%), and one physician (14.2%). Results from the qualitative interviews suggest that participants found the GWP had utility in three main domains: establishing and enhancing connection, satisfying basic needs, and promoting person-centered care.

#### Establishing and enhancing connection

In a population that faces marginalization, and as a result often holds a mistrust in health care systems and providers, the project offered a way to establish trust with the care team.

“I think it helps [the clients].. Sometimes it's hard to get these [clients] to trust us so I think when we do a really good thing like that it helps them see that we're not bad people. But not even that, that we care.” (Participant 5)

As a group that often faces social isolation, especially at end of life, and holds a well-founded fear of dying alone, the project fortified existing provider–client relationships and offered authentic human connection.

“Sometimes it's so busy … that sometimes the person gets lost in it … There's no time to connect … and [the GWP] offered that space. And now looking back, it made it easier for [the client] … it made our engagement more meaningful and it made [the client] believe that we cared.” (Participant 6)

#### Satisfying basic needs

Participants acknowledged how the wishes tended more toward necessity rather than luxury.

“… Everything is about sustenance and daily survival. And if there's a gift, it does not surprise me that most of our patients, or many of them, put these gifts towards a more practical use.” (Participant 2)

Seemingly “simple” wishes showcased just how resource limited the population was.

“When you're used to getting basic income, you sort of just do the bare minimum and all those other things that should be basic, don't end up becoming very basic for you.” (Participant 1)

The project offered an avenue to provide these “basic needs.”

“It built bridges for individuals to access resources that I believe that would otherwise be non-existent for them—or very very hard to access.” (Participant 3)

#### Promoting person-centered care

Wish exploration and granting allowed participants to go beyond the “medical agenda,” better understand their clients as individuals, and thus provide care that acknowledges their personhood.

“We're able to be more creative because we can ask those questions to them… When you ask it in the form of a wish you realize how important it is to them… you can put in more effort to support.” (Participant 1)

Participants acknowledged the hardships and dehumanization that the homeless and vulnerably housed face and how the project offered a means to celebrate them as unique and valued individuals.

“These clients have had difficult lives, not to say that other people haven't, but you know you're often feeling neglected or not worthy of care and support and to be able to make them feel like they're a unique, special person is such a beautiful thing to witness.” (Participant 4)

A group that often falls into the gaps—the forgotten of society—were allowed to truly be seen.

“They became the visible ones.” (Participant 3)

#### Facilitators of success

Two key enablers of success were identified: sharing responsibility with the care team and, in turn, the project being rewarding for those providers involved. Although wish elicitation and granting were assigned to the GWP team, participants found that involving the care team in wish exploration and implementation helped clients establish trust with the GWP team and proved to be more timely and effective.

“I think organizationally we want it to be detached from the care providers but I think realistically it becomes part of what we're doing as a team. Because we make that referral, because of the connection they have with us.” (Participant 1)

Participants also described how personally gratifying it was to be involved within the project.

“When you're doing day-to-day role it's one thing, but when you get to do something that you know will make them smile… something that [they] wanted, and you're part of that? It's really rewarding. It doesn't feel like you're doing something extra. It feels like you should have done this all along.” (Participant 1)

#### Barriers to success and recommendations

In adapting the 3WP to this client population, some challenges were identified. Many participants were unclear around the criteria for a “wish” and thus felt uncertain when engaging with clients around their wishes.

“I'd like to have more information to know what it can be and what it can't be so I could support clients in helping them determine what their wish could be.” (Participant 4)

From this feedback came the recommendation to establish clear criteria for acceptable wishes to be shared with the care team.

Participants identified that some wishes were not able to be granted by the GWP team in a timely manner, which resulted in some clients dying before receiving their wish or receiving a gift that they were not able to utilize as their functional status had declined by the time they received it. As a result, shortly after the project started, the estimated prognosis required to be eligible for the project was lengthened from 3–6 to 12 months.

Participants identified that having a third-party team independently facilitating the project was a barrier in this client group who often face difficulty navigating systems and establishing trust.

“If we have a difficult time getting in touch with them and supporting them in the services that we provide, it's going to be difficult for another, outside agency to get involved as well in building trust and all the stuff that we've already done.” (Participant 4)

Some participants were easily able to incorporate the project into their daily work, whereas others were investing significant amounts of time and going far beyond their regular workload.

“It wasn't easily incorporated into the schedule because it was a separate program from what I was hired to do… So it was quite difficult to incorporate it… there were numerous hours that I did spend, over and above.” (Participant 3)

From the mentioned challenges came the recommendation to increase the involvement of, and communication with, the care team to share the workload, assist in establishing trust and navigation of the project, and ultimately, to simplify the process. Ideally, there would be the addition of more staff or volunteers behind the scenes (e.g., finding, purchasing, and delivering gifts) to relieve the workload of the care team and to avoid taking time away from patient care.

## Discussion

Previous research calls for comprehensive, flexible, and creative solutions to address the gap in palliative care for individuals experiencing homelessness.^31,32^ Inspired by the 3WP,^27^ the GWP proposes an innovative intervention for enhancing the end-of-life experience for individuals experiencing homelessness. The project aimed to bring comfort and dignity to their final days by exploring and granting their wishes as a way to honor a population that faces hardship and marginalization.

The 3WP classified wishes into five categories: humanizing the environment, personal tributes, family reconnections, rituals and observances, and paying-it-forward.^27^ Many of these wishes looked quite different from those seen in the GWP, which centered more around practicalities (e.g., clothing and groceries), yet similarly to the 3WP, focused on comfort, connection, and altruism. There are multiple factors that may account for these differences. In the 3WP, wishes were elicited from patients, family members, or their clinicians; whereas in the GWP, wishes were elicited from the patients themselves. In addition, the 3WP was performed in an ICU where patients were imminently dying and likely had limited functional statuses, whereas the GWP was in a community setting with broader prognoses. Although both groups may experience dehumanization at end of life, the nature of this experience significantly differs between groups. In the 3WP, the acknowledged dehumanization comes as a result of the ICU setting, whereas, for the homeless population, this dehumanization stems from enduring experiences of extreme poverty, stigma, and structural violence.

Providers involved in the care of GWP clients identified how the project enhanced their ability to see the individual person beyond their diagnosis and circumstances and increased the personal rewards of caregiving. In addition, it offered a means of establishing trusting relationships, which are known to be important for the provision of quality palliative care to those experiencing homelessness.^20^ Exploring and granting wishes at an earlier stage in their illness trajectory not only allowed for the care team to better understand the client's values and goals, but also allowed for delivery of wishes at a time when the clients could still enjoy and appreciate those wishes.

In addition to the 3WP, there are several other published evaluations of the impact of wish-granting programs at end of life. All of these focus on children. Improvement in physical and psychological symptoms was demonstrated as compared with control groups.^33,34^ Quality-of-life measures improved in both studies, as did positive emotions such as hope. A case control study has also demonstrated a decrease in health care utilization for children participating in a wish-granting program.^35^ Besides the obvious population differences between adults and children, the children's studies looked at fulfillment of “grand wishes” of the children and often involved significant resources in contrast to both the GWP and the 3WP. A key research question is whether smaller scale wish fulfillment for adults can produce the same positive outcomes as those programs studied for children.

A challenge in replicating the GWP is that it requires ongoing funding and utilizes a partnering organization. This may make it difficult to adapt to other groups or populations that lack similar infrastructure. However, the scale-up experience of the 3WP demonstrated that this can be possible.^28^ A key learning of the scale-up experience of the 3WP, with relevance to our population, is the importance of local adaptation and a team approach to care.^28^

### Limitations

Two main limitations of the methodology were identified. A single researcher coded the responses without benefit of validation by a second coder. Second, the utility of the project was gleaned from the perspective of its providers rather than the clients themselves. It was decided not to interview clients due to the potential burden this would impose on them at a difficult time of their lives. Further research should focus on the client-centered experience and measuring quality-of-life outcomes.

## Conclusion

The GWP is a promising psychosocial intervention to improve the quality of palliative care for individuals experiencing homelessness, whose lives have largely been burdened with hardship and marginalization. In a population that often holds a mistrust in health care systems and providers, the project offers a way to establish trust with the care team and enhances authentic human connection. It also offered a way to acknowledge personhood, prioritize the clients' agendas, and celebrate them as individuals. The wishes showcase the scarcity of resources this population has and offers a means to satisfy unmet basic needs. Although interventions like these are important to address gaps and provide equitable care to populations that face barriers in access to palliative care services, we must at the same time acknowledge and address the social, structural, and societal factors that create these inequities in the first place.

## Supplementary Material

Supplemental data

## Supplementary Material

Supplementary Appendix SA1

## Abbreviations Used

3WP: 3 Wishes Project
GWP: Good Wishes Project
ICHA: Inner City Health Associates
ICU: intensive care unit
PEACH: Palliative Education and Care for the Homeless
