# Supplementary material for: The Good Wishes Project: An End-of-Life Intervention for Individuals Experiencing Homelessness
Source: Palliat Med Rep. 2020 Nov 18;1(1):264–9. doi: 10.1089/pmr.2020.0006 (PMC8241317; doi:10.1089/pmr.2020.0006)
Supplement: Supplemental data [file Supp_Data.pdf]

Supplementary Material

Supplementary Appendix SA1. Semistructured Interview Guide

- In what professional role did you interact with clients of the Good Wishes Project (GWP)?
- Were you a member of the GWP team or the Palliative Education and Care for the Homeless (PEACH) team?
- In what setting(s) did you interact with clients of the GWP?
- How many clients of the GWP have you been involved in caring for?
- Can you describe your experience with the GWP?
- The goal of the GWP was to provide dignity at the end of life through personalizing the end-of-life experience for patients who are living in homelessness or are vulnerably housed. Do you think the program achieved this?
  - If so, what enabled it to succeed?
  - If not, why not?
- What were some challenges with the program?
- How much time did you personally dedicate to the GWP per week, on average? Was this work easily incorporated into your working schedule or did you find you were spending extra time beyond your regular schedule to assist with the project?
- [Only applicable for clinical staff] How did it affect the care you as a care provider provided?
- Did the program affect the relationship you have/had with the clients?
- Did the program present any dilemmas for you personally, the clients in the program, or for the PEACH program? If yes, can you tell us about these dilemmas?
- In your opinion, were there any harms associated with the project? For example:
  - Were there any instances wherein you believe the project negatively impacted a participant or the care they received? Negatively impacted their friends and/or family?
  - Were there any undesirable consequences to your participation in this project or harms to you as a provider secondary to your participation?
- Do you have any recommendations for improvements to the program?
- Would you recommend the project continue? Why or why not?
